# Evaluation of Parental Acceptability and Use of Intervention Components to Reduce Pre-School Children’s Intake of Sugar-Rich Food and Drinks

**DOI:** 10.3390/ijerph19137967

**Published:** 2022-06-29

**Authors:** Bodil Just Christensen, Sidse Marie Sidenius Bestle, Ellen Trolle, Anja Pia Biltoft-Jensen, Jeppe Matthiessen, Sarah Jegsmark Gibbons, Anne Dahl Lassen

**Affiliations:** National Food Institute, Technical University of Denmark, Kemitorvet Building 201, 2800 Kongens Lyngby, Denmark; simsib@food.dtu.dk (S.M.S.B.); eltr@food.dtu.dk (E.T.); apbj@food.dtu.dk (A.P.B.-J.); jmat@food.dtu.dk (J.M.); sajegi@food.dtu.dk (S.J.G.); adla@food.dtu.dk (A.D.L.)

**Keywords:** discretionary food, family-based intervention, school health nurse, social cognitive theory, dietary guidelines, pre-school children, qualitative interviews

## Abstract

Knowledge is needed about effective tools that reach public health objectives focused on reducing the intake of sugar-rich foods and drinks. The purpose of this study was to assess the parental acceptability, use and motivational potential of intervention components developed in the randomized family-based trial ‘Are you too sweet?’ aimed at reducing the intake of sugar-rich foods and drinks among children (5–7 y). Intervention components included guidance on sugar-rich foods and drinks at a school health nurse consultation, a box with home-use materials and a digital platform. The methods used were a questionnaire among intervention families (*n* = 83) and semi-structured interviews with parents in selected intervention families (*n* = 24). Results showed the good acceptability and usefulness of the components, with reported frequencies of use of materials ranging from 48% to 94% and a high satisfaction rate with the school health nurse consultation. Personalized feedback and guidance from the school health nurse seemed to be a motivational trigger, and components that were compatible with existing practices were most frequently used. However, the components were not considered engaging by all families. Overall, intervention components were well received and hold the potential for enhancing parental knowledge and parenting practices regarding limiting the intake of sugar-rich foods and drinks.

## 1. Introduction

Danish children and adolescents are too sweet in the sense that their average intake of sugar-rich foods and drinks exceeds the recommended maximum amounts [1]. This challenge is not limited to Denmark, as the pattern holds across Western countries [2,3], though Denmark holds the title of ‘world champions’ in buying sugar confectionery [4]. Studies have shown that the intake pattern of sugar-rich foods and drinks in childhood track into adulthood [5] and cause an elevated risk for dental caries and nutrient dilution, and the literature shows an association between a high intake of sugar-rich drinks and the risk of obesity, cardio-vascular diseases, type 2 diabetes and certain forms of cancer [6,7,8,9].

In order to reach public health objectives that are focused on reducing the intake of sugar-rich foods and drinks, knowledge of effective intervention components and strategies is needed. Despite a substantial number of studies and reviews on improving dietary behaviours, interventions that target children’s excess intake of sugar-rich foods and drinks are sparse. Few studies focus on sugar-rich foods (see, e.g., [10,11]), while more studies focus on sugar-rich drinks [12,13,14,15,16], often with an emphasis on environmental changes [17]. However, there is a lack of evidence synthesis to guide practice [18,19]. Equally, few reviews mapping efficient intervention designs or effective intervention components and tools to reduce the intake of sugar-rich foods and drinks exist; Johnson et al. describe how observational studies have linked restrictive parental feeding practices, such as coercive control or pressure, with higher intakes of sugar-rich foods and drinks among children aged 4–8 years. Furthermore, frequent television use is associated with higher intakes of sugar-rich foods, but effective intervention strategies are not yet systematically identified [3]. Likewise, Grieger et al. conclude that studies are required to assess the effectiveness of strategies identified in their review, i.e., reformulation, substitution, restriction/elimination, supplementation and nutrition education/messages [2].

Whereas evidence for effective reduction strategies is lacking, several studies have shown that both parental style and parental dietary practices are decisive concerning young children’s eating patterns [20,21]. A recent study among pre-school children and their parents found that the parents’ food-related practices (behaviours such as food rules, snack routines, restrictions, and nutrition education) have a greater influence on health behaviours than parental style (parents’ general parenting approach, either authoritative, authoritarian, indulgent, or uninvolved) [22]. The authors encourage the development of tools in future interventions and programs that improve and strengthen parenting practices as it holds important potential for health promotion [22]. The family-based approach is further supported by results showing that early establishment of healthy dietary patterns seems to be effective as it promotes health both during childhood and later in life [23].

A number of behavioural and practical barriers have been found in relation to parental behaviour change on dietary habits [24,25]. In regard to sugar-rich foods and drinks, studies have shown that a widespread lack of knowledge among parents on portion sizes and maximum intake among children is a major impediment to behavioural change [26,27]. Another recurrent barrier to change is parental non-commitment to or rejection of recommendations [28,29]. Interventions that advise parents to change how much (portion sizes) or what (e.g., sugar-rich foods and drinks) they serve to their children necessarily involve the emotionally sensitive subject of parenting [30]. The challenge is to give dietary advice that builds knowledge and creates motivation for change without judging or blaming the current parenting. Previous studies have shown that interventions that aim to change dietary habits carry the risk of offending parents, as intervention content such as campaign messages, recommendations, education materials, tools, or other resources are inevitably normative and might leave parents feeling implicitly judged or blamed for their child’s diet and eating patterns [31,32]. As the acceptability of intervention messages and components is crucial for their effectiveness and probability of implementation, insights into how parents receive dietary advice addressed to their children are imperative in the development of interventions that aim to engage and support families to change their dietary habits.

In line with these previous findings, the present study aims to evaluate the acceptability and use of the intervention components developed in the intervention study “Are you too sweet?”, where Danish pre-school children aged 5–7 years and their families were enrolled [33]. The goal of the intervention was to decrease children’s intake of sugar-rich foods and drinks by increasing knowledge, motivation and self-efficacy in families. School health nurses were chosen as the mode of delivery as the consultation provided a personalized, in-person mode anchored in an organizationally structured frame [34]. Further, school health nurses are highly qualified in health education, motivational interviewing and engaging parents [35] and provide an opportunity to reach all children and their families regardless of social background [36]. In addition to the consultation, the intervention components included a box with a range of knowledge-building and behaviour support materials supplemented by a private Facebook group.

Based on an approach combining questionnaire responses and qualitative interviews, this study reports parents’ perceptions and use of the ‘Are you too sweet?’ intervention components and tools. The main aim of the study was to evaluate the acceptability and motivational potential of the intervention components. Moreover, the study aimed to elucidate if the components increased the behavioural capability for behaviour change and if specific intervention messages or components were experienced as patronizing or offensive.

## 2. Materials and Methods

### 2.1. Setting and Intervention Design

The 3.5-month intervention study ‘Are you too sweet?’ was performed in the Danish municipality of Hvidovre. The municipality was chosen because it is close to the national mean for socio-economic status, ethnicity, and education level in Denmark. Informed by the socio-economic index scores used in the municipality, six schools were selected to participate in the intervention study. The index scores were a continuous variable calculated on the basis of parents’ income, marital status, ethnicity, etc. The schools were cluster-randomized to be either intervention (*n* = 4) or control schools (*n* = 2). A detailed description of the study design has been published previously [33]. The intervention was conducted from late fall 2020 to early spring 2021 during the COVID-19 pandemic, with several school classes closing for single or several weeks with short notice. The baseline and follow-up measurements, however, were conducted as planned with few modifications (e.g., online interviews).

In short, the intervention components included an extended consultation with the school health nurse with an increased focus on the child’s intake of sugar-rich foods and drinks, including feedback from a short web-based assessment tool, the sugar-rich food screener (see Section 2.2). A box with home-use materials was handed out by the school nurses aiming to engage and inspire the families to decrease the intake of sugar-rich foods and drinks (see Section 2.3), and finally, the parents were offered to participate in a private Facebook group during the intervention period (see Section 2.3).

Social cognitive theory was the guiding framework for the intervention design and components. The main aim was to increase knowledge, motivation, behavioural capability and self-efficacy and thereby secure the prerequisites for behaviour change [33]. In order to address the inherent risk of patronizing and to secure the development of non-offensive behaviour change strategies and intervention components, a set of formative research measures were undertaken in the development process. The research elements were informed by parenting theories on tailoring intrinsic motivational messages [37] and encompassed 10 preparatory qualitative interviews with parents to identify value-based or contextual barriers; two focus group interviews to assess acceptability of intervention messages, selected components and delivery mode; and a pilot study with eight families to test feasibility and acceptability. Interviews and tests led to progressive modifications and adjustments to, e.g., the design of components and message phrasing in order to minimize the inherent risk of rejection (of, e.g., the new guidelines on sugar-rich foods) and avoid any tendency to preach ‘correct parenting’ or give parents the impression that they were receiving a lecture.

The acceptability and usefulness of the intervention components were evaluated by questionnaire responses from 83 families from an evaluation section in the follow-up questionnaire, combined with 24 semi-structured interviews with participating families evaluating their perceptions and practices concerning the ‘Are you too sweet?’ intervention components. Two focus group interviews with participating school health nurses have been analyzed previously to capture their experience with intervention components [38].

### 2.2. Consultation with the School Health Nurse, New Guidelines and the Sugar-Rich Food Screener

**Figure 1:**
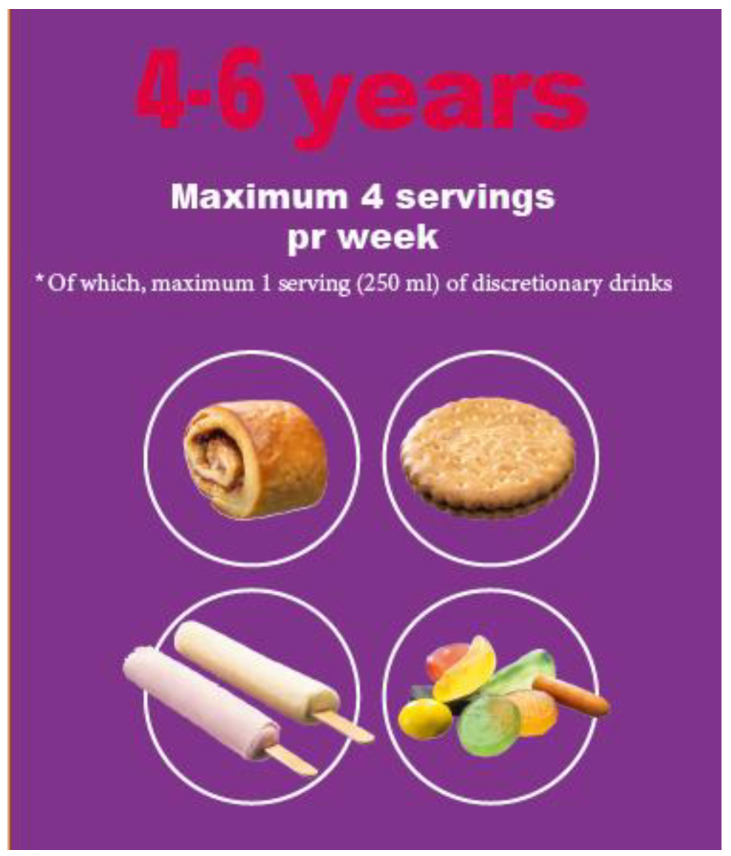
The maximum intake advised for 4–6-year-old children is four weekly servings per week consisting of 450 kJ of sugar-rich foods, * of which, maximum one serving (250 mL) of discretionary drinks.

A key element in the ‘Are you too sweet?’ intervention was the consultation with the school health nurse as a setting for communicating the newly developed maximum limits on discretionary food and drink intake [39], with discretionary foods and drinks being defined as sugar-sweetened and artificially sweetened beverages, sweets, chocolate, biscuits, ice cream, pastries, cakes, salty snacks and other energy-dense, nutrient-poor foods [1]. The maximum intake advised for 4–6-year-old children is four weekly servings consisting of 450 kJ of discretionary foods, equivalent to, e.g., one sandwich cookie, one small cinnamon roll, two lollies and 30 g of gummy bears or similar pick ’n’ mix sweets (Figure 1). The definition and development of guidelines for discretionary foods and drinks have been described in more detail elsewhere [1].

**Figure 2:**
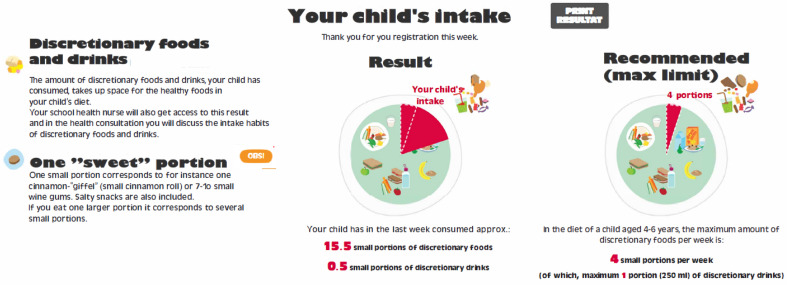
The output from the sugar-rich food screener displaying, as an example, the intake of, in all, 16 small portions (15.5 small portions of discretionary foods and 0.5 portions of discretionary drinks) in the left pie chart and the recommended weekly maximum number of small portions (four small portions) in the right. To the left is the definition of portions. In the original output, the text is in Danish but is translated to English here for better comprehension.

All families, including both parents and the enrolled child, were invited to the consultation with the school health nurse. The consultation unfolded as a conversation on everyday routines and family life related to well-being and health and was guided by a conversation tool prompting the core topics of diet, physical activity, screen time, sleep and well-being. The consultation is a mandatory practice in Danish pre-school but was extended from 30 to 35 min, where the additional five minutes were dedicated to discussing the intake and eating habits of sugar-rich foods and drinks. As part of the intervention and as preparation for the consultation, a short web-based assessment tool, ‘the sugar-rich food screener’, was developed to assess the intake of sugar-rich foods and drinks prior to the health consultation at the school. The tool was subsequently validated [40]. Intervention families received a link and were asked to fill out the ‘sugar-rich food screener’ three days prior to the consultation and were to register how much sugar-rich foods and drinks their child ate and drank over the past seven days. The intake of sugar-rich foods and drinks registered in the screener was visualized as an individual output displaying the number of sweet servings the child had consumed and the share that the sugar-rich foods and drinks took up from staple foods. See Figure 2 for an example.

Further, the school health nurses had access to a text summary of the discretionary food intake. Portion sizes and practical and social context of the intake were included, and this information enabled the nurses to tailor an informed conversation with the family about the child’s intake habits and discuss current practices and potential changes in habits to better health.

### 2.3. Box with Home-Use Materials and Facebook Community

The box with home-use materials contained the following materials: a serving size board illustrating the maximum amount of servings of sugar-rich foods and drinks in a recommended diet with reusable stickers with different examples of servings of cookies, chocolate, ice cream etc.; an inspiration booklet describing different strategies to curb sugar habits; an educational card game (the Monster Game); pamphlets with suggestions for local family activities; a read-aloud children’s book; and three small posters and stickers with the project logo. All materials except the children’s book and the pamphlets were developed for the intervention. Supplementary to the home-use toolkit, intervention families had access to an educational app with two learning games and an augmented reality function (AR-function) and were invited to subscribe to a private Facebook group used to provide parents with information and ‘reminders’ of the project during the intervention period. The Facebook group was designed as an opportunity to build social support among the participating families (peers), as the group’s content was only visible to its members. For a more detailed description of the intervention components and their theoretical underpinnings of behaviour change strategies and determinants, see Bestle et al., 2020 [33].

### 2.4. Qualitative Interviews and Quantitative Questionnaire

A combination of methods was chosen, including a quantitative evaluation by a questionnaire directed at the parents to get an overall measure of the use of and satisfaction with the ‘Are you too sweet?’ intervention components and a qualitative evaluation from interviews of parents from selected families to get a deeper understanding of parental perceptions of and experiences with the components.

The follow-up questionnaire (post-intervention) comprised an evaluation section with 27 questions on the participants’ experiences, use, and satisfaction with the intervention components, the school health nurse consultation and the sugar-rich food screener. Frequency of use and satisfaction were evaluated using five-point Likert scale questions with response options ranging from ‘not used’ to ‘used more than five times’, and ‘very satisfied’ to ‘very dissatisfied’, respectively. These options were supplemented by a ‘don’t know/not relevant’ option. A total of 83 responses were obtained among the 89 participating families (response rate of 93%).

Post-intervention, 24 families were recruited for a qualitative evaluation interview. Interviews were conducted from two to six weeks after the end of the intervention. In order to recruit an adequate yet socio-economically representative sample, families from all four intervention schools were recruited by phone through random sampling. To reach the sample size, 35 families were contacted. Among the 11 families who declined the interview, the most common reason was lack of time.

The interviews were semi-structured, and a topic guide with open-ended questions was used (see Appendix A). Questions were supplemented by structured follow-up prompts and unstructured probes [41]. Themes included knowledge about and implementation of new guidelines on sugar-rich foods and drinks in the family, use of and assessment of the intervention components and the family’s practices around and perceptions of family time and values, food and notably sugar-rich foods and drinks. Due to the analytical focus on acceptability, feelings of blame and rationales for potential rejection of the intervention components were further explored since content, framings or designs that were experienced as offensive by the participants would constitute the main barrier to implementation and behaviour change.

All interviews were conducted by B.J.C. with either one or two parents from each family online due to COVID-19 restrictions. Following oral consent, interviewees were provided with a link to a Microsoft Teams Meeting set up by the interviewer, and interviews were recorded using the Microsoft Teams video conferencing software (Microsoft. Redmond, Washington, DC, USA). Interviews averaged 61 min in length. Recordings were subsequently transcribed verbatim as text documents.

### 2.5. Data Analysis

Results from interviews were obtained by an iterative thematic approach that was applied to the 24 interviews using the framework of thematic content analysis [42]. Through an inductive, open-coding strategy, a preliminary coding framework was developed by two researchers. The double coder approach was employed to increase quality and ensure the identification of a broad range of themes and to utilize the differences in proposed codes as a resource, thereby enhancing the refinement of the coding framework. To establish coding reliability, the procedures proposed by Campbell were used, first determining the units of analysis, then ‘blinding’ them and subsequently applying codes [43]. The first reliability test resulted in 77% agreement, a result that led to the refinement of the coding scheme and an ensuing second reliability test. The second test provided 86% agreement and was evaluated as satisfactory, as it corresponded to the suggested standard of 80–95% agreement, though there is no universally accepted threshold for what indicates acceptable reliability [44]. In all, five interviews (21% of the sample) were reviewed to determine reliability between the two coders. Subsequently, coding of all transcripts was conducted by the primary researcher (B.J.C.) using NVivo software version 10, (QSR International, Doncaster, Australia). The questionnaire survey was conducted using LimeSurvey version 3.15.5+, (LimeSurvey GmbH, Hamburg, Germany). Descriptive and frequency summaries were computed in Excel for responses to each of the 27 questions.

## 3. Results

### 3.1. Participant Characteristics

Table 1 details the main characteristics of the 24 interviewed families and the total intervention population for comparison. There was a fair representation of parents of girls and boys, and the distribution of parental educational background among the interviewees resembled the sample distribution.

### 3.2. Perception of the Consultation with the School Health Nurse and the Sugar-Rich Food Screener Output

In the questionnaire responses, the majority of the 83 families indicated that they were either satisfied (53%) or very satisfied (28%) with the consultation with the school health nurse. No respondents indicated that they were dissatisfied with the consultation.

In the interviews, two main profiles of parents were identified in the analysis regarding the presentation of the sugar-rich food screener output and the ensuing advice at the school health nurse consultation. One profile was composed of parents who considered the consultation as ‘fine’ or ‘a cozy chat’, but did not deem it to have any significant impact on their perception of their own health habits or their child’s intake of sugar-rich foods and drinks. Parents in this profile accounted for around one-third of the interviewees.

*“Well, I must admit, I actually do not really remember what the health nurse said”, father to girl at school A.*

The other profile accounted for a larger part of the interview sample and consisted of parents who conveyed that the consultation and ensuing advice had a substantial impact on their perception of the family’s sugar habits. Several interviewees reported that they had experienced the sugar-rich food screener output as an ‘eye-opener’ and hence a ‘wake-up call’ to reduce their child’s intake of sugar-rich foods and drinks.

*“I would say we were probably both in shock because we believe we have a healthy relationship with sweets, so we were very surprised”, mother to girl at school D.*

Several parents further explained that their astonishment was caused by the fact that their child’s intake was much higher than they expected and markedly higher than the maximum number of weekly servings in a recommended diet; this was information that considerably changed their image of themselves as having a healthy diet and their perception of their family’s sugar habits as being well-balanced and reasonable.

*“I was damn proud when we signed up and I thought "we totally got this" and then when we got that pie chart (from the sugar-rich food screener), I was kind of like "oh, okay… the higher you fly, the further you fall”, father to girl at school D.*

The novel and, for some interviewees, disquieting information on the guidelines in combination with knowledge on their child’s intake in relation to these guidelines served as a cue to action and spurred most parents to consider possible changes. The guidelines and the school health nurse’s explanations were reported to have had a high motivational impact on parents to follow the advice and guidance.

*“I acknowledged her point when she had drawn it in red, which means alarm. Then you think "mayday-mayday." We need to do something”, mother to boy at school B.*

As mentioned, one of the aims of the consultation was to encourage families to change their habits related to the high intake of sugar-rich foods and drinks. Interviewees explained how they experienced the conversation and the behaviour change suggestions from the school health nurse as helpful and relevant.

*“We also had a chat about how it matters to change the little things. It is not like we were supposed to go home and change everything. That is not at all what it is about. But yes, (reducing our intake of) squash may be a good place to start. What would be good alternatives to that, right?”, mother to girl at school D.*

A sub-theme that emerged was the consideration of the unhealthy impact of sugar-rich foods and drinks other than weight gain. Some of the parents explained that before the intervention, they did not consider limiting their child’s intake as long as the child did not have an unhealthy weight development. However, the visualization in the sugar-rich food screener output revealing how a diet that fills up on sugar-rich foods and drinks provides less nutritional value to the child’s body made them reconsider their practice.

*“I was surprised it was an issue since he is so skinny. But then again, you also talk about the inside of the body and whether it consists of muscle or fat. So, I still listened, even though I was offended at first”, mother to boy at school B.*

Interviewees also expressed that the fact that the consultation with the school health nurse, which both encompassed parents and the child, had a decisive impact on the subsequent behaviour changes at home. Several parents reported that the child was more compliant and positively received the health messages and guidelines on maximum intakes as the advice came from the school health nurse. Hence, parents could refer to the school health nurse as a trusted sender and thereby encourage the child to be mindful of what they had learned during the consultation.

*“She (interviewee’s daughter) knew very well that “okay it was not just mom”, mother to girl at school C.*

Across the interviewees from both profiles, it was reported that the personalized feedback and adjustment of advice provided by the school health nurse made the guidelines more relevant and relatable.

Several parents expressed that their child’s intake of sugar-rich foods and drinks somewhat or largely exceeded the advised maximum servings in a recommended diet, and the parents’ astonishment over how little room for sweet treats the guidelines allowed for was a recurrent theme in the interviews.

*“And then the four pieces of candy for her age. That seemed a bit grotesque. I was like "Wow! That is hardly anything!", father to girl at school C.*

Despite their amazement, parents stated that they perceived the guidelines as useful and motivating in reducing their child’s intake of sugar-rich foods and drinks.

### 3.3. The Acceptability and Use of the Intervention Components Used at Home

#### 3.3.1. Quantitative Evaluation

Table 2 shows participating families´ frequency of use of the home-use intervention components. The inspiration booklet and the read-aloud children’s book were the most-used components of the home-use materials (used by 94% and 81%, respectively, once or more). Additional questions (not shown) revealed that the most common use of the inspiration booklet was either to use the booklet as a conversation starter in the family (40%) or to get new knowledge and inspiration (27%). The serving size board with reusable stickers and the educational card game (the Monster Game) were used by about two-thirds of the families (used by 59% and 63%, respectively, once or more). The main reasons for not trying out the card game were that families either forgot (30%) or did not manage to get it done (33%) (data not shown). The least-used component was the educational app, which was used by less than half of the families (48%). The most common reason not to download the app was that families forgot (62%), while others had technical difficulties (12%) or other difficulties (6%) (data not shown).

Table 3 shows that among those using the home-use materials, the majority expressed that they were either satisfied or very satisfied with the components (65–85%), except for the educational card game (the Monster Game), where only around half of the users expressed that they were either satisfied or very satisfied with the component (50%).

With regard to the Facebook option, 61% answered that one or both parents had subscribed (data not shown). Results on satisfaction revealed that most of the subscribers were neither satisfied nor dissatisfied (40%) with this component, whereas around one-third expressed that they were either satisfied or very satisfied with it (37%). Additional questions (not shown) revealed that less than half (46%) had posted, liked, or commented in the group. When asked about the lacking interaction, subscribers rated the content as relevant (95%) but indicated that they did not know what to comment or post (31%) or that they rarely interact on Facebook.

In the following, parents’ perceptions and use of the home-use materials and digital resources will be described one by one by evaluations drawn from the qualitative interviews.

#### 3.3.2. Serving Size Board with Reusable Stickers

In the interviews, families who used the serving size board all agreed that the concrete imagery was an effective way to communicate the guidelines and portion sizes. Whether families have used the board to plan for or ‘keep accounts’ of sugar-rich food and drink intake, the board has been the joint point of reference for the parents and the child.

*“It still hangs out there on the fridge (…) it has worked well because it has been an actual visual thing at her eye level, right. And it has been noticeable in the kitchen, and we could say: “But look. Now you are asking for this, but you already have two stickers, and it is only Friday tomorrow"”, mother to girl at school C.*

In this way, the serving size board functioned as a tangible and easy-to-understand tool to explain the portion sizes and the maximum number of weekly servings for the child, thereby making the child assist in the monitoring and management of the intake of sugar-rich foods and drinks.

Some parents expressed that they had used the board to make the child aware of serving sizes but without combining it with the guidelines and the number of maximum servings. In other families, notably, the stickers had been turned into a random toy, but with no explicit health message or educational purpose.

*“And then there were the stickers. They have used them in all sorts of funny ways (laughs), but that is probably just a kid’s thing”, mother to boy at school A.*

Not all families used the serving size board or the stickers to monitor intake. Some expressed that they found the logic of counting or planning sweet servings irrelevant to their practices as they perceived the guidelines as a general frame for healthy eating and did not follow the guidelines for the limited number of servings. Others had a more value-based rejection of the serving size board, as it was directly aimed at the child as a monitoring tool for their intake. Parents believed that it should not be the child’s concern to understand and comply with the guidelines, e.g., the maximum number of four weekly servings (see Figure 1) and therefore rejected the tool.

*“It is just, that thing about a six-year-old having to comprehend what she can and cannot have. Well, listen up! The idea is that we present the food she needs. And that is the proper food. Nothing more! And if you eat what we present, then we believe you will get some healthy habits”, father to girl at school C.*

For them, decision-making on food choice was a parental responsibility, not to be conferred to pre-school children who were thereby rendered individually responsible.

*“I believe it is my responsibility. Not his”, mother to boy at school A.*

A recurrent critique expressed among parents who disapprove of the responsibilization of the child through the serving size board was the separation of foodstuff into ‘allowed’ and ‘allowed in limited amount’ categories, and thereby ‘good’ and ‘bad’ foods. Parents expressed that conceptualizing food in these categorical ways, in their opinion, paved the way for a dichotomous health talk that they did not want to induce in their child as they believed it could imbue feelings of guilt and anxiety.

*“It is very important to me to teach them good habits, so that they learn to make reasonable choices, I mean, do away with this idea of prohibited or bad foods”, mother to girl at school D.*

Parents stated that *because* health literacy was important, they were cautious. In their approach, health was a fine line to walk, and it could unintentionally be disrupted and result in adverse consequences that may be serious and irreversible, e.g., disturbed eating [45]. They believed that with age, children should build the ability to navigate and handle the complex demarcation lines between healthy and unhealthy, but only later.

It is important to underline that these parents did not necessarily disapprove of the guidelines as such but criticized the transfer of responsibility for monitoring the intake to the child that the serving size board conveyed. To them, this task required a thorough, nuanced knowledge of nutrition in order to make balanced choices.

#### 3.3.3. The Inspiration Booklet

Families generally conceived the booklet as useful information, easy to access and a resource to give a summary of the guidelines and the background. Among the parents who had used it, some had studied the themes and ideas for changing family sugar habits in more detail and used it as a go-to resource; others had briefly flipped it through and considered to which extent the knowledge and strategies were useful for them.

*“I thought it was nice to receive those tips and tricks because it made me look them up again. Like, "what was the message again?"”, mother to girl at school B.*

Notably, the use of the booklet as a reference for definitions and guidance was underscored, as the booklet was used as a resource that parents could return to when in doubt. Several parents mentioned the opening with different examples of 450 kJ servings as particularly helpful.

*“It is this one (shows the servings sizes in the inspiration booklet) I think that is the one we have used the most”, mother to girl at school C.*

Some parents explained that they already knew several of the strategies for reducing intake or background information on healthy eating already, but in combination with knowledge of the guidelines, it assured them that their rules and routines around sugar-rich foods and drinks were ‘sensible’ and essentially in line with the guidelines.

*“To me it was a good inspiration booklet. I probably just needed that service check of our habits, "what are we doing?" and it helped me”, mother to girl at school C.*

A few participants used the booklet to establish a common understanding with, e.g., their partner, or they had asked grandparents to read it in order for them to obtain knowledge on the guidelines and advice.

*“Not long ago I told my husband to read it as well, so we are in it, like, together”, mother to girl at school B.*

Results thus indicate that for most parents, the booklet served as a helpful reminder both of the guidelines and, e.g., servings sizes, and of strategies and advice of which they knew the majority before they enrolled in the project.

#### 3.3.4. Educational Card Game (the Monster Game)

The educational card game, the Monster Game, is a deck of cards that can be used for two different games and combined with the augmented reality (AR) monster that comes to life when stickers with invisible QR-codes are scanned with, e.g., a smartphone or a tablet. The gameplay was designed to be played as a matching game or in a storytelling version, enabling reflections on habits and intake of sweet foods and drinks in the family where stickers could be placed at strategically chosen spots in the home.

The interview data displayed that families who had played the game overall liked it. Only a few used the second option of the card game, where cards were used to engender dialogue about sugar habits among family members and to explore their own preferences and routines and potential strategies to reduce the intake of sugar-rich foods and drinks.

*“We used the game a couple of times. We have not played the actual game a lot. We have been more like making up the stories. We used that part of it, the one with making up a true and a false story. And then the part with thinking of alternatives, because it was actually the kids just as much as myself who came up with the idea of having Friday fruit”, mother to girl at school C.*

As the box with home-use materials in many families was framed as belonging to the child, the child was likewise ‘the manager’ of the card game, and some parents explained how the child had invented personal rules or used the cards according to rules pertaining to other card games.

*“He loves flipping lottery. I tried to explain to him what we were supposed to do and stuff, but in the end we made it a flipping game instead”, mother to boy at school A.*

The impact of the educational value of the card game differed among families; while some children did not ascribe any particular meaning to the green cards with ‘healthy foods’ and the red cards with ‘unhealthy foods’, others took away an understanding of the (relatively simple) health message behind the gameplay. However, several parents questioned the card game’s capacity to successfully promote learning and development.

*“I was initially assuming that the kids were to learn about sweets and healthiness and stuff like that, but that was not at all what they were taught. Focus was on capturing the monster and learning how to capture it”, mother to girl at school A.*

Several families never got started with the game, either because the child (or their sibling) did not want to play, because the parents experienced the gameplay as too complex, because they had lost the manual or similar reasons. The most common reason was the rulebook being too complicated or time-consuming to read.

*“There were too many rules. There was, like, too much to comprehend”, father to boy at school C.*

Others simply did not find the time or forgot about the card game.

*“We never really looked at it. It was somehow just forgotten among everything else”, mother to girl at school B.*

The intervention ran during the Danish COVID-19 lockdown, and the particular circumstances constraining everyday routines impacted family life in general. Families explain how time was an (extra) scarce resource and that parental educational ambitions were lowered.

*“I would definitely have spent more time on the game, had it been more of a usual everyday life, as that would also mean more time for it. In the current situation we need to stick to the familiar”, mother to girl at school A.*

Data thus indicate that because the card game demanded preparation time and engagement from parents, the card game was not played in several families.

#### 3.3.5. Read Aloud Children’s Book ‘Anton Og Sukkerdillen’

Almost all families participating in the interviews had read the book, ‘Anton og Sukkerdillen’, aloud to their child and often also read it to the child’s siblings.

*“[The book] was funny. They really liked it, her little sister as well, also in relation to dentists and such. It is really good”, mother to girl at school A.*

Many parents explained how the family’s bedtime routine includes reading aloud and that children choose which book to read. For some, ‘Anton og Sukkerdillen’ became one among other popular books, while other children got less involved with the story or preferred other genres.

*“He likes to choose which books to read. It is not one he has asked for”, mother to boy at school A.*

The book’s health education message concerns dental care and the importance of a balanced diet and reducing the intake frequency of sugar-rich foods and drinks. How the health promotion message was received differed among families, as it was evident to some, but not to others.

*“We have read it a couple of times at least. But, like, I think they see it as a story just like any other”, mother to girl at school B.*

*“The thing with the teeth falling out and "do you remember the crocodile who just suddenly had no teeth". So yes, they got it. It did make an impression on them”, mother to girl at school D.*

Few families did not read the book, mainly due to practical impediments and not disapproval. Parents’ feedback indicated that the easy adaptability of the book into current practices and bedtime routines is a crucial element of its successful implementation in families’ everyday life.

#### 3.3.6. Educational App with Learning Games and AR-Function

These evaluations were mirrored in the interviews, where parents of children who have used the app assessed that the health education message was easy to grasp and children liked the gamification concept.

*“He liked the app; the one where you can feed it with lots of sugar, or greens and then it, like, got better or did not get better. He thought that was funny. Yeah, and then the fact that it could talk to him”, mother to boy at school A.*

The evaluation of the app from the child’s perspective differed widely and determined the frequency of use.

*“Then we tried that app. He did not find that interesting, the one where those gizmos jump around. He really thought that was boring”, mother to boy at school A.*

As with the card game, the educational app demanded an initial effort from parents to install the app and explain the functionality to the child. In some families, this was an impediment to use. For others, technical challenges prohibited it from being downloaded. To some, technical issues became an insurmountable obstacle due to general frustration with online platforms and digital resources related to the COVID-19 lockdown.

*“I must say, with all this lockdown and corona. It has been incredible with this home-school ing craze and all that technical shit and stuff. So, having to download an app and figuring it out. (tired sigh!) I was just very “no!””, father to girl at school C.*

This argument also conveyed the general situation characterized by a lack of time and energy that many families reported and thus not a critique of the app as such.

#### 3.3.7. Private Facebook Group

As the survey data showed, most subscribers were inactive; they did not post or comment on posts from the project team, even when different engagement tactics were employed by the administrators (who were part of the intervention team). In the interviews, participants could clarify and give more details on the lack of activity among subscribers.

*“I do not use Facebook for communication purposes. I simply use it as a tool to look into what people are doing. To probe into people’s lives (laughs)”, father to girl at school C.*

Some parents did not subscribe either because they missed the invitation or because they had dropped social media out of principle, but for parents who subscribed, the evaluation of the group was positive. For the most part, they liked the content but just did not want to comment or like, simply because they rarely interacted on social media. When asked, parents explained that the topics of health, dietary patterns and parental care were sensitive, and they were hesitant to discuss them with, e.g., fellow parents that they hardly knew.

*“I probably would have done it in another setting where I knew who the members were and then I probably would have chosen the Facebook group that belongs to (child’s name) class. So, like a slightly narrower forum. I only used the Facebook group for inspiration or information. So, only as something for me, not something from me”, mother to girl at school C.*

Though very few parents contributed actively with content or commentaries, many read the posts that the project team wrote and posted on a regular basis. They received the notifications, and for many, the posts worked as a welcome reminder.

*“Yes, but it was nice to have ongoing reminders, because you can easily forget all about it and then get back on the wrong track. Starting again to buy candy, even if you really do not want to. "Why did I do this? There is no reason to do so. ” So it was a really good reminder”, mother to girl at school A.*

Results showed that the Facebook group did not unfold as planned with regard to providing social interaction, but subscribers reported that content and notifications worked as helpful reminders and instigated motivation and engagement.

#### 3.3.8. The Child-Centered Approach as a Basis for a Shared Language

As a crosscutting theme concerning several components, interviewees highlighted the all-family approach in the communication of the guidelines present in both the serving size board, the inspiration booklet, the educational card game (the Monster Game) and the learning games in the app. As the one-by-one presentations have shown, these learning resources included a range of child-oriented, visual, and easy-to-understand tools developed to explain the guidelines. Participants evaluated them as being very useful. The tools equipped them with applicable arguments and logic when discussing reductions and rules on sweet treats with their child.

*“She understands if we show her: "At your age you should not have more than this". And then she can more easily put it into perspective, and, like, really understand and accept it”, mother to girl at school B.*

The parent-child materials provided guidance to help parents explain the guidelines. This shared language on sugar-rich foods and drinks was reported to have helped with making the child understand why reducing the intake of sugar-rich foods and drinks was important and had improved the quality and nuance of the conversation that the family had concerning their sweet habits.

*“We have just discussed it: "But there are simply no biscuits for now because listen, you have four available, and therefore you can have an apple"”, mother to girl at school C.*

However, as described in relation to the serving size board, not all parents agreed upon introducing this intervention tool to their child and adopted an adult-centered approach as a conscious strategy.

## 4. Discussion

This study showed an overall good parental acceptability of the intervention components in the family-based intervention “Are you too sweet?” aiming at reducing the intake of sugar-rich foods and drinks among children. The key modality for message delivery was new guidelines on sugar-rich foods and drinks [1] communicated to the families through a consultation with the school health nurse, including individual registration and output through ‘the sugar-rich food screener’, supplemented by a box with the home-use materials and a private Facebook group to support parenting practices around limiting the intake of sugar-rich foods and drinks.

While all families attended the school health nurse consultation and, in general, expressed satisfaction with both the consultation and the individual registration and output from the ‘sugar-rich food screener’, both the questionnaire responses and the analysis of the qualitative interviews showed an uneven frequency of use of the home-use materials and, likewise, a certain degree of variation in their satisfaction rating. No component was deemed offensive or inadequate, but not equally relevant or useful either. As a general pattern, components that demanded little effort and were compatible with existing practices were more easily implemented and more frequently used, e.g., the inspiration booklet and the read-aloud children’s book, while the Monster Game and educational app provided as a part of the home-use materials were used by fewer families and in general with less satisfaction.

### 4.1. School Health Nurse Consultation and the Sugar-Rich Food Screener

A main component in the ‘Are you too sweet?’ intervention was the communication of the developed maximum limits on sugar-rich foods and drinks at the school health nurse consultation and the associated individually tailored advice. Families’ evaluation emphasized the school health nurse as a trusted information sender, notably for the children. This is in line with another qualitative study on the experience of school health nurses working with overweight children in elementary schools in Sweden, where the nurses’ sensitivity to individual needs and ability to provide individual support and advice was considered to be pivotal [46]. Further, in combination with the consultation set-up encompassing both parents and child, the consultation was mentioned as important for establishing the foundation for a shared language on sugar-rich foods and drinks for some.

Participants underscored the usefulness of the personalized guidance in regard to the family’s habits and actual intakes. The in-person individual feedback made information relevant and relatable. The differentiated guidance was enabled by the ‘sugar-rich food screener’, and results showed that the screener equally functioned as a motivational trigger for many parents as the individualized feedback and visualization of the maximum weekly servings displayed the consequences of a high intake in a tangible and easy-to-grasp manner. In a preceding evaluation conducted among the participating school health nurses, they expressed their satisfaction with the information on individual intakes and actual habits that the sugar-rich food screener provided, which allowed them to tailor advice to the family’s specific needs [38]. Other studies support how and why the tailoring of advice increases self-efficacy and behavioural capability by providing participants with the knowledge and tools necessary to set and pursue their goals [47,48,49]. The high acceptability indicates that the sugar-rich food screener and the interpretation of the output by an educated health advisor (the school health nurse) are efficient and that the ‘Are you too sweet?’ team has succeeded in designing a tool that may improve engagement and self-efficacy. It should be underscored, however, that though most parents reported an outcome of the health dialogue with the school health nurse, some parents seemed to benefit less as they found the guidelines and advice less relevant despite the individualized approach. The stance points to a much-debated dilemma in public health ethics: the conflict between the potential paternalistic effects of intervention and individual autonomy [50]; or, as Riiser has asked: “can we justify imposing on the participant’s personal preferences by directing actions for his or her own good?” [51] (p. 241).

### 4.2. Components and Materials Used at Home

The box with home-use materials that families received included a serving-size board with reusable stickers, an inspiration booklet, an educational card game (the Monster Game), a read-aloud children’s book, and access to an educational app with learning games. In addition, parents were invited to subscribe to a private Facebook group. Responses from the questionnaire showed a certain degree of variation in the use of the home-use materials. While the inspiration booklet and the read-aloud children’s book were looked through or read by most participating families (94% and 82%, respectively), about two-thirds of the families used the serving size board and card game (62% and 65%, respectively), and around half of the participants used the educational app (49%). With regard to the Facebook group, around four out of six participants subscribed. Among the participants who had used the materials, the same degree of variation was found in their satisfaction ratings. Participants that were either satisfied or very satisfied ranged from 35% and 53% for the Facebook group and the educational card game, respectively, to 74% and 86% for the serving-size board and the read-aloud children’s book. Hence, some components seem more accessible to participants than others, a finding that is mirrored in the interview data, where families report that components that demanded preparation, such as downloading an app or reading rule books or where, e.g., technical difficulty with initial set-up could occur, were less likely to be used. This corresponds to findings from other studies using games and apps [52] that describe poor usability in relation to, e.g., non-intuitive interfaces or technical obstacles. These impediments might have been an even greater obstacle to overcome due to the COVID-19 context, where many parents experienced distress and a lack of time and resources due to the imposed additional work strain of juggling the challenges of home-schooling (often of more than one child) while working remotely themselves. In relation to the Facebook group, the distress and other contextual effects of the societal lockdown in Denmark might likewise explain the frequent assessment of the group and its function as ‘a kind reminder’. Despite the lack of social interaction, the Facebook group thus indirectly instigated motivation and engagement. Other studies evaluating behaviour change and motivational techniques in interventions support the effectiveness of digital prompts as cues to reinforce motivation and potentially behaviour change [11,53]. The findings describe how prompts, e.g., in push notifications, increase parental engagement and that parents find the content helpful [53].

Parents who used the intervention components expressed that their behavioural capability increased through the educational properties of notably the booklet and the serving size board. In the interview data, interviewees emphasized the serving size board as a good tool to convey the guidelines to their children and that the stickers were used to cue serving sizes and maximum intakes. Results from the interviews showed that a fraction of parents did not use the serving size board (and were therefore not asked to evaluate it in the questionnaire) because they did not approve of what they deemed a potential responsibilization embedded in the design. In addition, some objected to the division of foods into ‘allowed’ and ‘allowed in limited amount’ categories, and thereby ‘good’ and ‘bad’ foods. This finding underlines the importance of communicating healthy eating messages that emphasize a balance of food and drinks and avoiding an exaggerated focus on single foods when introducing the components to families.

However, as the serving size board was not imposed as mandatory but offered as an optional tool, parents who disapproved of it could easily refrain from using it. The board still holds a capacity for transfer of responsibility whereby the child is rendered individually responsible for intake pattern or monitoring of intake in relation to the guidelines. The statements in the interviews from the sub-group of parents disapproving of the responsibilization are important in this regard, notably because these same parents, in general, approve of the guidelines as such. Their disapproval of the serving size board expressed in the interviews underlines the unavoidable, inherent risk of responsibilization in child-oriented intervention components that seek to enhance health literacy in the child. A responsibilization of the child could cause feelings of pressure and guilt that might engender negative social and emotional experiences around food and eating. Several studies have shown how such experiences might lead to less healthy eating habits [3,54]. In other families, the child-oriented components facilitated a shared language on sugar-rich foods and drinks and thus invited co-management and collaborative decisions on, e.g., intake patterns. Such practices hold the potential for a transfer of responsibility to the child but do not necessarily induce it. The balance between responsibilization and increased health literacy in the child is a fine line, and the interviewees navigated it differently due to their diverse parenting values.

When assessing the home-use materials in combination, families did not universally prefer one (type of) material. The diverse modalities were each favoured and combined differently from family to family, and it might be argued that the range of different modalities allowed families to customize their own selection of tools and resources to tailor ‘their family intervention’. Evaluated against the aim of empowering and motivating participants to generate their own new healthier habits, this is positive.

### 4.3. Engagement of Families Regarding the Intake of Sugar-Rich Foods and Drinks

Considering the feedback from the consultation with the school health nurse, where several parents relayed that it did not have any significant impact on their perception of their own health habits or their child’s intake of sugar-rich foods and drinks, it might be questioned to what extent the intervention components were increasing engagement universally. The evaluation of the materials and tools might be positive, and acceptability might be high, but this may not instigate changes among all families as not all parents are motivated and accordingly not compelled to engage in any behaviour change. If the intervention message of reducing the intake of sugar-rich foods and drinks does not align with parental core values, the aim for increased motivation for change will not be attainable, as motivation is conditioned by concordance with personal beliefs and core values [37].

In studies aiming to explain modest results of dietary interventions, insufficient effects are often attributed to social barriers and a lack of specificity or resources [55,56,57]. Moreover, health promotion campaigns and interventions inevitably raise ethical issues as they demarcate normative standards for ‘correct behaviour’ [58]. Parenting studies have furthermore stipulated the risks of evoking negative emotional responses among parents when correcting their current dietary practices [30,31]. The ‘Are you too sweet?’ study aimed to overcome these barriers by offering diverse strategies and a motivationally driven range of intervention components to engage and empower families. Overall, results suggest that the ‘Are you too sweet?’ project team largely achieved the aim of developing a useful, empowering, and, in general, non-offensive toolkit. However, the aim of engaging all families seems not to have been achieved.

### 4.4. Strength and Limitations

It is a strength that questionnaire data was obtained from 83 of 89 participating families (93%) and that 24 families were interviewed. This provides detailed data material for the analyses. Additionally, it is a strength that a broad spectrum of socio-economic levels among participants was obtained and that the study population thus covers a diverse selection of family types and socio-economic statuses. Fathers were still under-represented despite the efforts to recruit them. Furthermore, the study could have been made more nuanced by interviewing the children alongside the parents in the evaluation interview [59], in the same way as in-person interviews would have been favoured to the online version imposed by the pandemic.

It is a strength of the study that the range of different intervention components allowed families to customize their own selection of tools and resources according to preferences; however, a consequence of this is that the intervention components cannot be evaluated separately. In addition, it was not explicitly evaluated if participants conceived of the recommended maximum number of weekly servings as comprising both salty and sweet discretionary foods [1]. As mentioned throughout the article, the impact of the COVID-19 pandemic, lockdowns and related changes in the everyday life of the families might have influenced their participation in the intervention, but the effect is difficult to measure and thus adjust for. Families were differently affected depending on, e.g., their socio-economic situation and work-life organization. An additional limitation is the lack of observational data from the school health nurse consultations. Such data would have provided information on the nurses’ attitudes vis-a-vis the guidelines, their use of the intervention components and potential encouragement to use (selected) materials, as well as the strategies implemented to tailor advice to individual families. Such information would have enabled a more nuanced evaluation of the context for and impact of the consultation.

## 5. Conclusions

Results suggest that future initiatives to promote a reduced intake of sugar-rich foods and drinks among pre-schools should include individually tailored advice in accordance with parenting values. Knowledge-building materials might prove effective if combined with support tools for behaviour change. Intervention components were generally acceptable and non-offensive and had the potential to increase knowledge and behavioural capability and thereby strengthen parenting practices. The personalized feedback on intake in relation to the guidelines facilitated by school health nurses seemed to be a motivational trigger that made, notably, the knowledge-building and behaviour support materials relevant for many, but not all parents. Further, the intervention components were useful for parents as resources facilitating the translation of advice from the school health nurse into daily family practices, in particular when the component could be implemented in existing practices and routines. A sub-group of parents approved of the guidelines but did not use the serving size board, as the latent risk of responsibilization embedded in its use conflicted with their parenting values. Bearing this in mind, the components hold important potential for health promotion around sugar-rich foods and drinks. Components may significantly improve parental knowledge, establish the foundation for a shared language on sugar-rich foods and drinks and enhance parenting practices around limiting the intake of sugar-rich foods and drinks.

## Figures and Tables

**Table 1:** Characteristics of the interviewees in the evaluation interviews compared with the questionnaire respondents, which equals the total intervention group.

	Interviewees, *n* = 24	Questionnaire Respondents, *n* = 83
**Sex of participating child; *n* (%)**		
**Girls**	14 (58%)	44 (50%) *
**Boys**	10 (42%)	44 (50%) *
**Interviewees; *n* (%)**		
**Mother**	17 (71%)	66 (80%)
**Father**	1 (4%)	16 (19%)
**Step-mother**	1(4%)	1 (1%)
**Both parents**	5 (21%)	-
**Parental education; *n* (%)**		
**Basic school (<12 years)**	3 (13%)	9 (11%)
**Upper secondary school (12 years)**	0 (0%)	3 (4%)
**Vocational education (13 years, practical)**	7 (29%)	19 (23%)
**Short higher (13–14 years)**	4 (17%)	8 (10%)
**Medium higher (15–16 years)**	5 (21%)	21 (25%)
**Long higher (≥ 17 years)**	5 (21%)	23 (28%)
**Ethnicity (maternal); *n* (%)**		
**Danish**	22 (92%)	76 (92%)
**Other western and non-western**	2 (8%)	7 (8%)

* The total number of participating children is *n* = 88 in the 83 families as the sample included 3 pairs of twins and 1 set of triplets.

**Table 2:** Frequency of use of materials among participating families.

*N* = 83	Not Used	Used 1–2 Times	Used 3–5 Times	Used ≥ 6 Times
**Serving size board with reusable stickers**	34 (41%)	21 (25%)	11 (13%)	17 (20%)
**The inspiration booklet**	5 (6%)	48 (58%)	18 (22%)	12 (14%)
**Educational card game, The Monster Game**	31 (37%)	32 (39%)	15 (18%)	5 (6%)
**Read aloud children’s book**	16 (19%)	30 (36%)	17 (20%)	20 (24%)
**Educational app with learning games and AR**	43 (52%)	14 (17%)	12 (14%)	14 (17%)

**Table 3:** Ratings of materials among *users*.

	*N*	Very Dissatisfied	Dissatisfied	Neither Satisfied Nor Dissatisfied	Satisfied	Very Satisfied
**Serving size board with reusable stickers**	49	0 (0%)	1 (2%)	12 (24%)	17 (35%)	19 (39%)
**The inspiration booklet**	74 *	0 (0%)	1 (1%)	22 (28%)	36 (46%)	15 (19%)
**Educational card game, the Monster Game**	51 **	2 (4%)	3 (6%)	20 (38%)	21 (40%)	5 (10%)
**Read aloud children’s book**	67	1 (1%)	1 (1%)	8 (12%)	28 (42%)	29 (43%)
**Educational app with learning games and AR**	40	1 (2%)	4 (10%)	8 (20%)	18 (45%)	9 (23%)
**Private Facebook group**	44 ***	0 (0%)	4 (8%)	21 (40%)	17 (33%)	2 (4%)
**School health nurse consultation**	80 ****	0 (0)%	3 (4%)	10 (12%)	44 (53%)	23 (28%)

* Four responders did not rate the inspiration booklet and answered ‘Don’t know’; these have been subtracted from the total number of users; ** one responder did not rate the card game and answered ‘Don’t know’; this has been subtracted from the total number of users; *** Eight respondents did not rate the Facebook group and answered ‘Don’t know’; these have been subtracted from the total number of users; **** Three respondents did not rate their perception of the school health nurse consultation; these have been subtracted from the total number of users.

## Data Availability

Not applicable.

## Appendix A

The topic guide for qualitative evaluation interview with families was developed to assess the usefulness and acceptability of the intervention components and mode of delivery, and the families’ actual behaviour changes in relation to sugar-rich foods and drinks, and the experienced facilitators and barriers for these changes. Additionally, parents’ perception of the guidelines on the maximum number of weekly servings and their motivation for and potential experiences with implementing them was assessed.

In this study, the aim was to evaluate acceptability, usefulness and motivational potential of the intervention components, and hence not all topics in the guide have been included in the analysis, but only those relevant for this analysis.

**Table:**

Theme	Content	Focal Points	Time Frame
Introduction	1. Introduce the aim of the interview 2. Inform about the structure and themes of the interview 3. Inform about anonymity (no individual identities will be disclosed in publications) 4. The audio recording is for internal use only (data protection) 6. Oral consent to participate 5. Do you have any questions?	Present the aim of the interview. Introduce the logic of qualitative interviews (that interviewee’s subjective experiences and perceptions are central, and that their point of view is necessarily right) Inform on confidentiality, withdrawal and data protection Obtain consent of participation	4 min
Overall evaluation	How was it to be part of the ’Are you too sweet?’ intervention?	Participant’s top-of-mind evaluation and thoughts?	3 min
Behaviour change and motivation	Have you changed any habits or practices? What did the habits and intake of sugar-rich foods and drinks look like in your family before you enrolled? **Prompts:** Friday night sweets, lunch packs, soft drinks and other sweet drinks, grandparents and afternoon snacks **Additional prompts:** Experiences with the 7 day dietary registration Parental agreement on e.g. rules, habits and/or changes Sustainability of new habits (Friday night sweets, lunch packs, soft drinks, portion sizes and/or frequency of servings)—promoters and barriers	Successful behaviour change in family habits and routines Experienced barriers, facilitators and motivations in relation to behaviour change.	10 min
Knowledge on the guidelines for sugar-rich foods and drinks	Did you acquire knowledge through the project that you did not possess beforehand? **Prompts**: weekly number of maximum servings, portion sizes, definitions of sugar-rich foods and drinks, other? What are your thought on the logic of a maximum number of servings? Did you aim to comply with the guidelines? Did you customize the guidelines? E.g., tailor the number of maximum weekly servings? Other? What are the challenges of complying with the guidelines, if any?	The applicability and relevance of the guidelines How do parents perceive of the recommended maximum number of weekly servings? Experienced barriers, facilitators and motivations in relation to reducing the intake of sugar-rich foods and drinks to the recommended maximum number of weekly servings	8 min
Consultation with the school health nurse	What are your thought on the consultation with the school health nurse? **Prompt:** Sugar-rich food screener output Which family members participated (child, mother, farther, both) in the consultation?	Perception of the consultation with the school health nurse Mode of delivery for main message and intervention components, including the sugar-rich food screener output	5 min
Intervention components and tools	If we look at the things in the home-use box with materials, what did you actually use? **Prompts:** The serving size board? The inspiration booklet? The read-aloud children’s book? Did [child’s name] enjoy it? Did he/she capture the educational health message on dental health? The card game (The Monster Game)? Did [child’s name] enjoy it? Did he/she capture the educational health message? Did you download the app? Have you/[child’s name] used it? Which features have you/[child’s name] used? Did you subscribe to the Facebook group? Did you get notifications on the posts posted? Have you read/seen the posts? What are your thoughts on the content? Did the posts serve as a reminder of your participation in the project?	Practical use of the intervention components, and potential tailoring or innovations Pedagogical usefulness of the intervention components Practical or technical impediments Disapproval or critique of the intervention components	10 min
The COVID-19 pandemic, lockdowns and restrictions	How did the COVID-19 pandemic and lockdown affect your family and everyday life? Do you think you would have been more or less involved in the project if lockdown had not happened? Prompt: E.g., used the things in the box with home-use materials more or less?	To what extent and how has the COVID-19 pandemic and lockdown been a barrier or a facilitator concerning behavior change?	8 min
Social support	Did you discuss the project with family or friends? **Prompts**: Other parents from [child’s name] class? Close family members, e.g., aunts, uncles or grandparents?	Family networks, core values and norms	4 min
Outro	Do you have anything to add? Things that we did not discuss that are relevant to the evaluation? Do you have any questions?		3 min
